# Carbon Dioxide Angiography–Guided Ultrasound Renal Denervation in Refractory Hypertension With Chronic Kidney Disease

**DOI:** 10.1016/j.jaccas.2025.106043

**Published:** 2025-11-14

**Authors:** Mohamad Bahrou, Ahmed Shahab, Rami Al-Ayyubi, John Flack, Abdul Moiz Hafiz

**Affiliations:** a Department of Medicine, Internal Medicine Residency Training Program, Southern Illinois University School of Medicine, Springfield, Illinois, USA; b Division of Cardiology, Department of Medicine, Southern Illinois University School of Medicine, Springfield, Illinois, USA; c Division of General Internal Medicine–Hypertension Section, Department of Medicine and Population Science and Policy, Southern Illinois University School of Medicine, Springfield, Illinois, USA

**Keywords:** carbon dioxide angiography (CO_2_ angiography), chronic kidney disease (CKD), refractory hypertension, ultrasound renal denervation (uRDN)

## Abstract

**Background:**

Renal denervation (RDN) is an emerging therapy for resistant hypertension. However, use of iodinated contrast during the procedure raises concern for contrast-induced kidney injury in patients with chronic kidney disease (CKD). Carbon dioxide (CO_2_) angiography may provide a safer alternative.

**Case Summary:**

An 85-year-old man with stage 3a CKD and refractory hypertension despite 5 medications underwent ultrasound-guided RDN (uRDN). After minimal iodinated contrast for access confirmation, CO_2_ angiography was used to visualize the bilateral renal arteries and guide ablation. Three ablations were delivered to each artery, and the procedure was uneventful. At 5-week follow-up, serum creatinine improved (1.3 mg/dL), and antihypertensive therapy was reduced from 5 to 3 agents.

**Discussion:**

CO_2_ angiography allows safe renal artery visualization without nephrotoxic exposure, making it particularly valuable in CKD patients where contrast-induced injury is a concern.

**Take-Home Message:**

CO_2_-guided uRDN is a safe, non-nephrotoxic option for refractory hypertension in patients with CKD.

## Graphical Abstract

**Figure:**
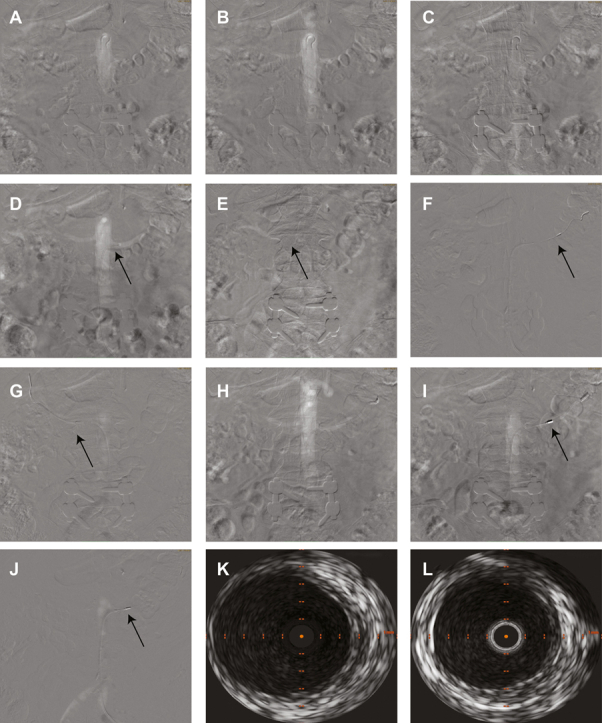

## Background

Renal denervation (RDN) is a treatment for resistant hypertension that typically requires iodinated contrast, which can risk kidney injury in patients with chronic kidney disease (CKD). Carbon dioxide (CO_2_) renal artery angiography offers a potentially safer imaging alternative during RDN for patients with CKD.^1^ We report to our knowledge the first successful case of CO_2_ angiography–guided, ultrasound-guided RDN (uRDN), performed in an older patient with refractory hypertension (prescribed 5 drugs at maximum tolerated doses, including a diuretic, yet blood pressure remained uncontrolled) and CKD.

**Take-Home Message**

• CO_2_ angiography–guided uRDN is a safe, feasible, non-nephrotoxic option for patients with CKD and refractory hypertension.

## Case Summary

An 85-year-old man with a medical history of long-standing, refractory hypertension, aortic atherosclerosis, stage 3a CKD, and hypothyroidism was referred for consideration of RDN as an adjunctive therapy to improve blood pressure control. His preprocedure blood pressure was 176/66 mm Hg. There was no direct or indirect evidence to suggest that he was nonadherent to his prescribed antihypertensive medications, which included amlodipine 10 mg twice daily, carvedilol 12.5 mg once daily, hydralazine 50 mg 3 times a day, spironolactone 25 mg once daily, and minoxidil 2.5 mg twice daily.

Laboratory studies revealed an estimated glomerular filtration rate (eGFR) of 45 mL/min/1.73 m^2^ and a serum creatinine of 1.5 mg/dL, consistent with stage 3a CKD. Thyroid-stimulating hormone levels were within normal limits on thyroid hormone replacement therapy. Transthoracic echocardiography demonstrated a preserved left ventricular ejection fraction of 66%, moderate concentric left ventricular hypertrophy, and grade 1 diastolic dysfunction.

**Figure 1:**
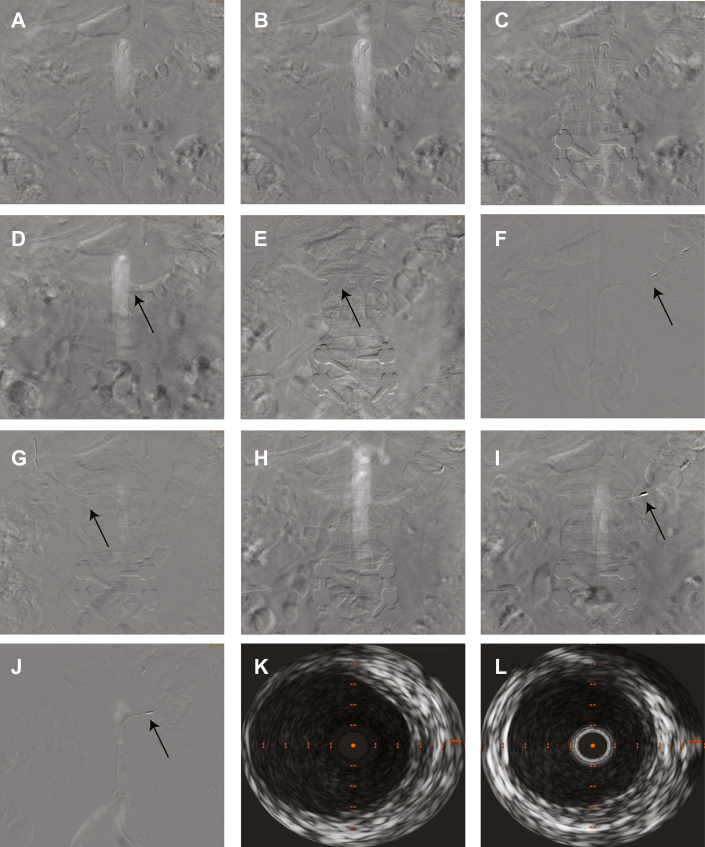
Aortograms, CO_2_ Angiography of the Renal Arteries, IVUS Imaging, and Pre- and Post-uRDN Imaging (A to C) Aortograms obtained using CO_2_ angiography revealing (A) the descending aorta and proximal sections of the bilateral renal arteries, (B) the descending aorta and proximal and mid sections of the bilateral renal arteries, and (C) the abdominal aorta and its bifurcation into the bilateral common iliac arteries. (D and E) Selective CO_2_ angiography of (D) the left renal artery and (E) the distal right renal artery showing distal bifurcation. (F to H) IVUS and post-uRDN imaging. IVUS images of (F) the left renal artery before uRDN therapy and (G) the right renal artery after uRDN therapy. (H) Aortogram obtained using CO_2_ angiography after uRDN therapy revealing the left renal artery with no aneurysmal changes or dissection. (I and J) Occlusion testing of the uRDN catheter at the (I) distal and (J) proximal left renal artery. (K and L) IVUS images of (K) the left renal artery before uRDN and (L) the right renal artery after uRDN. CO_2_ = carbon dioxide; IVUS = intravascular ultrasound; uRDN = ultrasound-guided renal denervation.

Given persistent refractory hypertension despite maximal tolerated medical therapy, the patient underwent RDN therapy after discussion and unanimous approval by a multidisciplinary Renal Denervation Eligibility Committee at our institution, which recommended using CO_2_ angiography. Left common femoral artery access was obtained, and access site angiography was performed with 10 mL of iodinated contrast followed by CO_2_ angiography of the abdominal aorta with runoff into the bilateral renal arteries. This visualization showed lack of atherosclerosis, stenosis, ostial lesions, or accessory renal artery branches. The left renal artery was cannulated using a renal double curve guide catheter, with dedicated CO_2_ angiography followed by intravascular ultrasound (IVUS) to assess vessel size and plan denervation sites. This was essential to ensure balloon and vessel 1:1 preferred sizing given that ultrasound energy delivery requires vessel wall contact, as well as to prevent vessel wall thermal injury during the cooling cycle of the device balloon before denervation occurs. The uRDN (Paradise uRDN system, Recor Medical) was performed at 3 nonoverlapping sites using an 8-mm balloon in the left renal artery and a 7-mm balloon in the right renal artery. Selective renal artery CO_2_ angiography was performed with the uRDN balloon inflated to confirm full vessel wall apposition before sonication. Postablation IVUS and imaging confirmed no dissection, perforation, residual air, or spasm (Figure 1). Vascular access site hemostasis was achieved, and the patient tolerated the procedure without complications.

The patient recovered well from the procedure and was discharged in stable condition the same day. Five weeks postprocedure, he reported feeling well. At his most recent clinic visit, his blood pressure was 170/54 mm Hg and serum creatinine was 1.3 mg/dL. His antihypertensive drug regimen was de-escalated, with removal of 2 of the 5 antihypertensive medications (minoxidil and hydralazine were stopped) and reduction in dosage on one of them (spironolactone was decreased to 12.5 mg).

## Discussion

Uncontrolled blood pressure in medically treated patients remains a significant concern, as blood pressure control rates for those prescribed medications is infrequently >70%. RDN therapy has emerged as a safe and clinically effective adjunctive therapy in multiple randomized sham-controlled trials,^2^, ^3^, ^4^, ^5^ and the procedure received U.S. Food & Drug Administration approval for patients with medically treated but uncontrolled blood pressure. Our patient manifested an extremely high-risk hypertension treatment failure phenotype, refractory hypertension, which has often simply been included as subset of resistant hypertension.

RDN has been evaluated as a therapeutic option for resistant hypertension in several clinical trials. The SYMPLICITY HTN-3 trial was the first large, randomized, sham-controlled study using radiofrequency ablation for RDN. However, it failed to meet its primary efficacy endpoint, showing no statistically significant reduction in blood pressure compared with the sham procedure at 6 months.^2^ In contrast, subsequent studies such as the SPYRAL HTN-OFF MED trial demonstrated significant reductions in blood pressure using radiofrequency-based RDN in patients with uncontrolled hypertension who were not taking antihypertensive medications.^3^ Similarly, the RADIANCE trials supported the efficacy of uRDN, also showing a significant blood pressure–lowering effect in patients with fewer added antihypertensives.^4^

However, in the landmark clinical RDN trials, patients with moderate to severe CKD were largely excluded or under-represented, and renal function cutoffs were chosen as safety parameters that have now become part of Food & Drug Administration labeling for procedure selection criteria. For example, the SPYRAL HTN-OFF MED trial excluded individuals with an eGFR of <45 mL/min/1.73 m^2^. Across the RADIANCE trials, patients with reduced eGFR of <60 mL/min/1.73 m^2^ were under-represented, comprising only 2.8% to 11.2% of participants, while excluding those with an eGFR of <40 mL/min/1.73 m^2^. Although the SYMPLICITY HTN-3 trial did not define a formal eGFR threshold, the study population had relatively preserved renal function.^2^, ^3^, ^4^

Similarly, the Paradise uRDN system is contraindicated in patients with an eGFR of <40 mL/min/1.73 m^2^.^6^ As a result, a critical gap remains in the current evidence regarding the safety and efficacy of RDN in patients with advanced CKD.

Established convention for RDN involves use of iodinated contrast agents to visualize the renal arteries. This poses an additional risk of inducing contrast-induced acute kidney injury, particularly in patients with CKD. CO_2_ angiography is a novel approach that is almost assuredly a safer alternative. It is characterized by its high solubility and lower density compared with iodinated contrast. It is injected into the renal arteries under fluoroscopic guidance, where it displaces blood and creates a negative contrast effect, allowing visualization of the vessel lumen. From our case experience, when combined with IVUS, this approach provides precise localization of renal artery anatomy, uRDN balloon placement planning, and confirmation of the absence of any vessel injury (such as dissection) postablation. CO_2_ angiography has previously been used for various diagnostic and interventional angiographic procedures where renal safety was a concern, including aortograms, central and wedged hepatic venography, renal angioplasty and stenting, catheter directed thrombolysis, as well as renal arteriography.^7^

The implementation of CO_2_ angiography for RDN is a novel approach, with only a few published reports of its use. Hameed et al^8^ evaluated the safety and efficacy of radiofrequency RDN using CO_2_ angiography in patients with moderate to severe CKD (eGFR: 15-44 mL/min/1.73 m^2^) and resistant hypertension. Eleven patients underwent the procedure and were followed for 6 months, demonstrating no significant decline in renal function, with a median serum creatinine difference of 0.25 mg/dL.^8^ Another prospective observational study by Lo et al^9^ evaluated radiofrequency RDN using CO_2_ angiography via radial access in 10 patients with resistant hypertension and moderate to severe CKD, with a mean baseline eGFR of 32.8 ± 9.4 mL/min/1.73 m^2^. Patients were followed for 3 months postprocedure and showed stable renal function, with a mean eGFR of 31.6 ± 10.2 mL/min/1.73 m^2^ at follow-up.^9^ There have not been any large randomized clinical trials or case reports examining CO_2_ angiography for uRDN to date.

There may also be a financial incentive for the use of CO_2_ angiography, as the cost of carbon dioxide is significantly lower than iodinated contrast, with no significant additional equipment cost.^10^ However, this might be offset by need to use IVUS, at least by operators who choose to use it. Whether combining IVUS and CO_2_ angiography is necessary is unclear at this time.

Based on our case, CO_2_ angiography appears to be a feasible alternative for patients with moderate CKD undergoing uRDN. Incorporating CO_2_ angiography into future uRDN trials could expand eligibility to high-risk CKD populations and generate more inclusive, real-world data on treatment safety and efficacy.

## Novelty of the Submission

To our knowledge, this is the first reported case of CO_2_ angiography–guided uRDN in a patient with refractory hypertension and stage 3a CKD, demonstrating its feasibility and safety as a non-nephrotoxic alternative to iodinated contrast.

## Conclusions

CO_2_ angiography–guided uRDN is potential non-nephrotoxic option for patients with medically treated but uncontrolled blood pressure and CKD. This approach minimizes the risk of contrast-induced acute kidney injury while maintaining procedural accuracy. However, more large-scale studies and case series are needed to further validate the long-term safety and efficacy of this approach.

## Funding Support and Author Disclosures

Funding for the open access publication fee was provided by Recor Medical. The authors have reported that they have no relationships relevant to the contents of this paper to disclose.
